# An Essay on the Treatment of Diseased Joints

**Published:** 1829-01-01

**Authors:** 


### XIII.

### AN ESSAY ON A NEW MODE OF TREATMENT FOR DISEASED JOINTS, AND THE NON-UNION OF FRACTURE; WITH CASES, AND FORMULÆ OF THE NEW PREPARATIONS USED. By *Thomas Buchanan*, C.M. &c. &c. Octavo, pp. 100. Longmans, London, 1828.

THE author of the above is favourably known to the profession, by several works of considerable merit. Hitherto, however, his essays have been upon the ear, his labours confined to a single division of surgical science, and the present is a bolder flight, we trust it will not turn out an Icarian one. Authorship is said to be a sin; certain it is, that authors invariably extenuate, or try to extenuate its commission by apologies of one kind or another. The following is Mr. Buchanan's.

"Several years ago, my attention was attracted to the extraordinary effects of the external application of the Tincture of Iodine, in the case of a patient whom I visited in the country. He had been attended in succession by two of the most eminent physicians in this town, along with the family surgeon. One of the physicians asserted that the disease was in the kidney, while the other as positively insisted on its being in the liver. Each of these gentlemen, in rotation, treated the case according to his own ideas of the seat of disease, and the indication of cure, without in any degree ameliorating the distressing situation of the patient, whose decease was daily expected. Such was the account I received when I saw him for the first time, being then attended by the family surgeon, the physicians having declared the case hopeless.

"The whole of the right hypochondriac region was enormously enlarged, so that when the patient lay on his left side, the parts projected, similar to that of the abdomen of a woman in the fourth month of pregnancy. From the appearance of the parts I was of the opinion that both the liver and kidney were diseased, particularly the latter.

"A singular circumstance was, that the patient had agreed for me to be sent for to receive instructions to inspect his body after death. I had however brought with me a small bottleful of the tincture of iodine, diluted with aqua calcis, and with the consent of the surgeon, and as a forlorn hope, applied it over all the parts diseased with a camel hair brush, to the extent of nearly half an ounce of this mixture, and left directions for this quantity to be applied in the same manner once every day. By following this mode of treatment, the patient was in a few weeks completely restored, and is at present pursuing all the laborious duties incident to the operative agriculturist, with ease to himself and advantage to his family. Encouraged by this almost unexpected cure, I began to apply the tincture externally to almost every case which resisted the ordinary routine of practice, and the result has been the production of the following pages." x.

After briefly summing up the various modes of treatment, recommended by all the most celebrated men who have written on the subject, our author proceeds to disclose his own, which, with him, "has produced resolution in the acute, and absorption of matter when formed in the chronic stage, without causing pain to the patient or injury to the system." Believing that the indication of cure consists in establishing healthy action in the parts diseased, and thereby alleviating pain and irritability, Mr. B. rejects, as inadmissible, blisters, issues, setons, and frictions, with or without liniments or ointments; inasmuch as they increase the local irritation. He also rejects the exhibition of medicine internally, because it must saturate and often disorder the system, before it affects the disease. Reasoning on the powers of mercury in inunction, the good effects of iodine in diseases of the ear, and the powers of the tincture, *locally* applied, in dispersing enlargements of the inguinal glands, our author was determined to make trial of the tincture, as a local application, in diseases of the joints. When a man has determined to execute a project, opportunities of doing so generally offer, at least it was the case with Mr. Buchanan. Within a few days of his making up his mind to employ the iodine, a middle-aged woman solicited advice.

The joint, between the first and second phalanx of the middle finger, had been wounded by a sickle eight days before, and was swelled to nearly twice its natural size. The wound had closed, and, as motion appeared to be lost, Mr. B. concluded that the tendon of the flexor muscle was divided, and had partially adhered. The tincture of iodine was applied to the part, by means of a camel-hair pencil, and the back of the hand being painful and swollen, the application was extended to the parts around. This treatment was repeated every morning—the swelling, in a few days, diminished—in eight days motion was partially restored—and the finger, at the end of a fortnight, was reduced to nearly its usual size, and rendered as useful as it was before the accident occurred.

The above was, in all probability, inflammation of the synovial membrane of the joint, and consequent effusion into its cavity. Whether Dame Nature or the tincture of iodine effected the cure, far be it from us to decide. Mr. Buchanan was confirmed in his opinion by the result, and employed the remedy in several cases, two more of which we shall notice. A seaman, æt. 27, just arrived from Hamburgh, presented himself with the left hand much swollen, the fingers so tumefied as not to allow ready motion, and the fore-arm also considerably swollen, with pain extending as high as the axilla. The back of the first phalanx of the little finger was laid bare by a wound, an inch in length, and a third of an inch in breadth, with elevated edges. The greater part of the muscular substance, surrounding the lateral and posterior parts of the phalanx to the joint, were detached from the bone, and presented a bluish colour near the periosteum. The tincture of iodine was applied to the tumid part, and the patient directed to take a mixture, containing calumba, quassia, and sulphate of magnesia, three times a-day. On the second day, the pain had subsided, and the swelling was diminished in the fore-arm and hand, but the joint of the finger was still enlarged. The wound was dressed with an ointment, composed of resin and acetic acid, spread on lint, the tincture applied in the wound and around it, and the finger bound up with a narrow roller. With the latter application daily continued, the patient was dismissed in six days more. A ship-mate, affected in a similar way, had suffered amputation of the hand, and died.

The following is a different description of case. A child, one year and nine months old, was brought to our author under the following circumstances. The right hip-joint was greatly enlarged—the limb was shortened—the toes inverted—the leg and thigh wasted. The appetite was bad, the little patient had hectic, was a twin, and had the signs of a scrofulous habit. The complaint had begun six months before, and was treated by leeches, poultices, and medicine, but without the most distant relief. One eminent physician refused to prescribe, alledging that the child could not survive the shock which the system had received. In addition to the symptoms first described, we should mention that a large collection of matter had formed over the posterior part of the joint. The integument presented a partial blush of red, and the abscess was apparently ready to burst. Great pain was produced on attempting to move, or even lightly touch, the limb. The tincture was applied, and a powder of carbonate of magnesia, dragon's blood, and rhubarb, given at nights. To these, a decoction of dulcamara was added; the treatment being pursued, with little alteration, till the end of five months, when the following report was made.

"The tincture has been applied every second day this week past. The muscular substance of the leg and thigh very much improved. During a considerable period no motion could be obtained without great pain. The application speedily caused a cessation of pain except when the joint was violently moved, and even then the pain was only partial. When absorption of the tumour took place, the parts continued for some time apparently of the same size, but turned gradually soft and spongy to the touch, and diminished almost imperceptibly.

"In the early period of the treatment, the integuments on, and around the joint, used to be more swollen some days than others; but now the parts are regularly of a uniform size, except a slight enlargement about the joint, and even this slight elevation is gradually diminishing." 45.

At present, 1828, the boy can run about without any assistance, and uses his limb with freedom and facility. A slight halt, however, remains, attributable, so Mr. B. thinks, to part of the head of the femur having been destroyed by ulceration before the tincture of iodine was used.

We fear that this new mode of treating diseases of the joints will scarcely prove of that paramount importance its advocate and author is sanguine enough to believe. Valeat quantum valere debet. Mr. Buchanan is far from confining the powers of the tincture to this class of complaints, extensive as it is. Gangrene, diseases of the spine, bubo, fistula, venereal nodes, inflammation of the mamma, and even non-uniting fractures, acknowledge the powers of the medicine.

"In the case of a strong healthy young man, who had his great toe crushed by a large heavy stone, which fell, and divided the toe at the second joint, the good effects of this mode of treatment was fully experienced. The parts were brought together and dressed, and two days afterwards gangrene took place; I had therefore, no other apparent resource, but either to apply a poultice to the parts until the slough was thrown off, or to amputate. In this dilemma I applied the tincture not only to the surrounding parts, but to the slough itself, and afterwards covered the wound with lint spread with the Ung. Resin. Comp. Part of the apparently dead substance of the toe became re-organized, so that instead of one half of the toe being thrown off, there was only a small piece of the skin detached; the bone united, parts healed and became as useful as before the accident happened." 49.

A young gentleman, of delicate constitution, applied with a very large venereal bubo, to which leeches and lotions had been frequently applied, without in the least arresting its progress. Mr. B. applied the tincture to the bubo, and also to an extent of more than two inches around it. On the third day, the pain and swelling were gone. Together with the iodine, the following was given, and sceptics might doubt to which the palm of victory belonged; six grains of blue pill, fifteen of jalap, and conserve of roses enough to make a bolus, taken at night.

"In two cases of fistula under my care, the one in the perinæum, and the other situated near the anus; the application of the tincture speedily effected a total obliteration of both the fistulous cavities. The fistula which was situated in the perinæum, had been an affection of several years standing, and of course required a longer period to effect a cure than the one near the anus. I have operated successfully for this complaint several times; but in future I should prefer the local application of the tincture, especially in the incipient stage, before it has communicated with the rectum or urethra, and have no doubt of effecting a cure in every instance.

"The external application of the tincture has, under my care, been of the greatest benefit in the discussion of nodes. A gentleman applied to me some time ago, under the most distressing circumstances. He had great pain in the scalp, particularly a little above the left orbit, and in the occipital region, where the parts were elevated, soft, and inflamed, with considerable depression in the cranium immediately underneath the sores, and easily perceptible to the touch. The pain was so intense, as to deprive him of sleep, and he was in the habit of taking a night draught of a hundred drops of the tincture of opium, and even plunging his head in water several times during the night, in order to afford a temporary suspension of the excruciating torture of this baneful disease.* Has been bled with leeches, cupped and blistered; and has used mercurial pills, ointments, &c. upwards of three years.

---

* "About twelve months prior to this period, the patient went to London ex-

"He was in this deplorable state when I first applied the tincture of Iodine to the nodes, in the manner described; in a few days a gradual cessation of pain took place, and in about five or six weeks, the integuments resumed their healthy appearance. The depressions became gradually filled with deposition of osseous substance, and he slept as sound as ever he was accustomed to do, prior to the beginning of this complaint. I prescribed at the same time, medicines for other symptoms of which he complained." 57.

In common inflammation and induration of the mamma, as well as in milk abscess and even in cases of cancer, Mr. Buchanan has derived the greatest advantage from the medicine. Several instances of cancerous affection of the breast were cured, at least the ulcers healed, whilst every symptom of disease disappeared, and none have yet returned. We now arrive at the second leading division of the work, headed, on the Treatment of Non-union of Fracture. After mentioning the practice of Hunter, who advised that the extremities of the bone should be rubbed together; of Mr. White, who excised the ends of bone; and, lastly, of Dr. Physic, who proposed and employed the seton, our author details a case where the tincture of iodine was used by himself.

"A seaman apprentice, ætatis 18, applied to me under the following circumstances. The patient had been employed during the preceding summer, on board of the ship Alfred, in the Davis' Straits fishery: and on the 31st of May his right leg was fractured by the tiller of the vessel, when she was making a '*stern board*' among the ice. The tibia and fibula were both broken, but reduced immediately afterwards by the surgeon of the vessel. The fracture being oblique, and bad weather occurring, the medical attendant failed in keeping the extremities of the bones in apposition. As to the propriety of his conduct, in allowing the bones to remain in that state, it forms no part of the present subject, and I shall therefore merely state the situation in which I found the limb at the time of application. The extremity of the lower portion of the fibula was detained in the gastrocnemius muscle, while the extremity of the upper portion was in partial contact with the extremity of the lower portion of the tibia, and the extremity of the upper portion of the tibia, from the obliquity of the fracture, overlayed, but in partial contact with the extremity of the lower portion. The patient was obliged to be supported, at first with a crutch, and afterwards with a staff, from the weakness of the limb, otherwise in excellent health, and a strong good looking young man. I applied a pledget of lint, dipped in a stimulating and astringent lotion, to the fracture, and then passed a bandage over it, from the toes to the knee, so as to cause pressure on the parts, and ordered him to take a wine glassful of the decoction of dulcamara three times a day. I persevered in this mode of treatment until April, 1827, but without the least success.

"Tired with this method, I proposed to cut down on the fracture, excise the extremities of the tibia and fibula, and then endeavour to place them in apposition. The owner of the vessel to whom the young man was apprentice, from principles of humanity, would not consent to the operation.

"On due reflection I was convinced, that if six months trial of the Hunterian mode, partially combined with that of Mr. Amesbury, had no effect in relieving any of the symptoms, they would not in all probability be any way relieved even by a continuance of seven years of the same mode of treatment.

"From the stimulating effects of the Tincture of Iodine when applied externally, I resolved to put its qualities to a severe test, by applying it to the limb, in the manner already described, in order to produce increased action of the arteries in the extremities of the fracture, and consequently secretion of ossific matter. At this

---

pressly for advice, and consulted the most eminent of the profession, and was at one time under the care of a gentleman attached to the medical department of His Majesty's Household, without experiencing any relief."

period it was with the greatest difficulty and extreme pain, that the patient could drag himself along with a staff. The limb was considerably swelled, particularly below the fracture; and if, when attempting to drag himself along, he touched a stone, or the least elevation above the common level of the path on which he walked, he was by that means put to the most excruciating torture. His foot, with the lower portion of the leg, could be wrought outwards and inwards, in a rotatory manner, when crepitus could be distinctly felt: while at the tame time, the knee and upper part of the leg were not affected by the motion of the foot. The limb was about two inches shorter than the other.

"*April* 16*th*. Applied the tincture to the limb, particularly about the fracture, and the parts around the ankle, and in three days afterwards, (19*th*,) the pain and swelling were removed. The patient took at the same time, a wine glassful of the Decoct. Dulcam. C. three times a day. I continued to apply the tincture every morning until May, and then applied it only every second day; the decoction was, however, continued as usual. The parts became stimulated, and deposited osseous substance, union of the extremities of the fractured bones took place, and in the month of August following, (1827) he was dismissed cured, with the limb apparently stronger than before the accident happened, and is now (1828) on board of his vessel, as active as formerly." 77.

With this we conclude our notice of the work, which should rather have been called a treatise on iodine than one on diseases of the joints. Mr. Buchanan undoubtedly merits commendation for the zeal he has displayed in his trials of the medicine, however divided opinion may be on the results. For our own parts, we believe the author has been led away by that leaning in its favour which all men must feel in pursuing a particular inquiry. The powers and qualities of iodine are far from being yet understood, and every contribution on the subject is therefore a matter of interest and importance. We have placed the main facts brought forward by our author on record, and leave the profession to judge of their value for itself.

